# Are the Efficacy and Safety of Chest Tubes in Uniportal Video-Assisted Thoracic Surgery Related to the Level of Intercostal Space Insertion or to the Drain Type? A Prospective Multicenter Study

**DOI:** 10.3390/jcm13020430

**Published:** 2024-01-12

**Authors:** Dania Nachira, Pietro Bertoglio, Mahmoud Ismail, Antonio Giulio Napolitano, Giuseppe Calabrese, Khrystyna Kuzmych, Maria Teresa Congedo, Carolina Sassorossi, Elisa Meacci, Leonardo Petracca Ciavarella, Marco Chiappetta, Filippo Lococo, Piergiorgio Solli, Stefano Margaritora

**Affiliations:** 1 Department of Thoracic Surgery, Fondazione Policlinico Universitario “A.Gemelli” IRCCS, Università Cattolica del Sacro Cuore, 00168 Rome, Italy; antoniogiulionapolitano@gmail.com (A.G.N.); giuseppe93calabrese@virgilio.it (G.C.); mariateresa.congedo@policlinicogemelli.it (M.T.C.); elisa.meacci@policlinicogemelli.it (E.M.); leonardo.petraccaciavarella@policlinicogemelli.it (L.P.C.); marco.chiappetta@policlinicogemelli.it (M.C.); filippo.lococo@policlinicogemelli.it (F.L.); stefano.margaritora@policlinicogemelli.it (S.M.); 2 Division of Thoracic Surgery, IRCCS Azienda Ospedaliero-Universitaria di Bologna, 40138 Bologna, Italy; 3 Division of Thoracic Surgery, Klinikum Ernst von Bergmann, Academic Hospital of the Charité-Universitätsmedizin, Humboldt University Berlin, 14467 Potsdam, Germany; mahmoud.ismail@klinikumevb.de

**Keywords:** uniportal VATS, biportal VATS, chest tube, smart coaxial drain, fluid output

## Abstract

Objectives: The aim of this study is to evaluate if the efficacy and safety of chest tube placement are influenced by the level of intercostal space insertion (uniportal VATS vs. biportal VATS) or by the type of drain employed (standard vs. smart coaxial drain). Methods: Data on patients who underwent either uniportal or biportal VATS upper lobectomies with lymphadenectomy were prospectively collected in three European centers. The uniportal VATS group with a 28 Fr standard chest tube (U-VATS standard) was compared with the uniportal VATS group with a 28 Fr smart drain (U-VATS smart), and U-VATS smart was also compared with biportal VATS with a 28 Fr smart drain inserted in the VIII intercostal space (Bi-VATS smart). Results: When comparing the U-VATS standard group with the U-VATS smart, a higher fluid output was recorded in the U-VATS smart (*p*: 0.004) in the III post-operative day (p.o.) and overall (*p*: 0.027), with a lower 90-day re-admission in the U-VATS smart (*p*: 0.04). The Bi-VATS smart group compared to U-VATS smart showed a higher fluid output in the I p.o. (*p* < 0.001), with no difference in total fluid amount or hospitalization. The Bi-VATS smart recorded a lower incidence (*p* < 0.001) of residual pleural space or effusion (*p*: 0.004) at chest X-rays prior to drain removal but a higher level of pain and chronic intercostal neuralgia (*p*: 0.03). Conclusions: Chest tube insertion through the same incision space in uniportal VATS seems to be safe and effective. Smart drains can improve the fluid output in uniportal VATS, as if the drainage were inserted in a lower space (i.e., biportal VATS), but with less discomfort.

## 1. Introduction

Traditionally, following a pulmonary lobectomy, the thoracic cavity is drained through two chest tubes: one positioned in the posterior basilar region of the chest cavity to facilitate fluid drainage, and the other one placed with its tip in the apex of the thorax to drain the air and improve lung re-expansion [1]. Due to post-operative pain and patient discomfort related to the two tubes, the introduction of video-assisted thoracic surgery (VATS) lobectomy changed the double chest drainage technique with the insertion of a single tube within the VII or VIII intercostal spaces. Several trials also showed the non-inferior efficacy of a single chest tube compared to two tubes in open and VATS (biportal or triportal) lobectomies [2,3,4].

In the last few years, uniportal VATS has further reduced the invasiveness of surgery to a single 3–4 cm incision approach, allowing for major lung resections [5,6,7,8,9]. In this technique, the chest tube is placed through the same surgical incision to avoid the irritation of other intercostal nerves, except that of the space used for the incision [7,8,9]. This trick seems to further reduce the onset of post-operative pain and improve patient recovery [10,11].

The incision in uniportal VATS is usually performed in the IV or V intercostal spaces along the middle axillary line [7,8,9]. Additionally, the insertion of a single chest tube through a high and antigravity space, straight to the apex of the thorax, is often criticized due to the possibility of inadequate drainage of the thoracic cavity, resulting in residual pleural effusion or insufficient lung re-expansion with residual pneumothorax. This may be more evident after upper lobectomies, where the risk of an empty pleural space in the apex is potentially higher and the necessity of good chest drainage becomes mandatory [12,13] to prevent any post-operative complications.

Therefore, while the efficacy and safety of the uniportal VATS technique for major lung resections were demonstrated in recent studies [9,14], the effectiveness of chest tube insertion through the same incision space is still debated due to the aforementioned potential risks [12,13].

Some studies [12,13,15] proposed possible solutions, such as the use of two chest tubes through the uniportal VATS incision [12], or the use of an additional pleural catheter (16 GA CVC) inserted in the VII/VIII intercostal spaces alone [12], or associated with the insertion of a single chest tube through the uniportal VATS incision [13].

Another solution is the employment of a modified T-tube, usually used in biliary duct surgery, for the simultaneous drainage of air and fluid [15].

**Figure 1:**
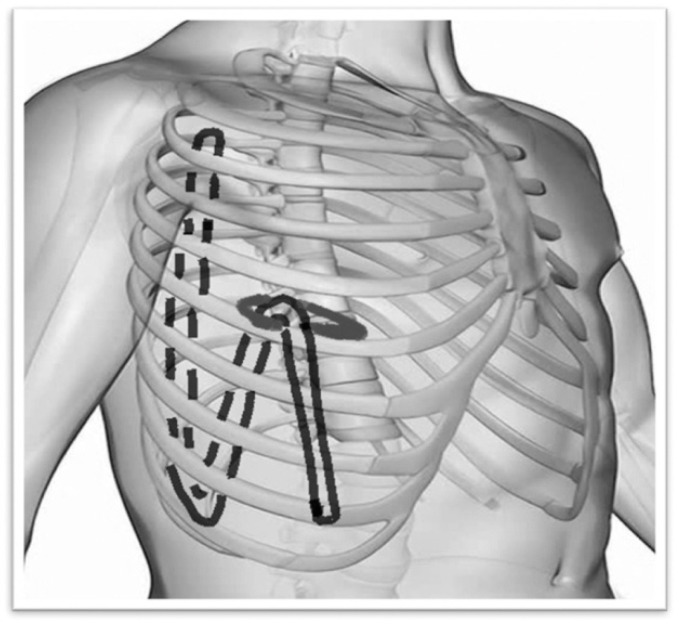
Suggested “S-shape” for standard chest tube in uniportal VATS, with its tip in the apex of the chest posteriorly and the curved part above the diaphragm.

In our uniportal VATS experience [9], two potential solutions were developed. The first involved the use of a standard 28 Fr chest tube inserted through the single surgical incision in such a manner as to create a particular “S-shape” during lung inflation, with its tip placed in the apex of the chest posteriorly and the curved part above the diaphragm (Figure 1) to optimize the drainage of both air and fluid, taking into consideration the patient’s varying decubitus and position.

**Figure 2:**
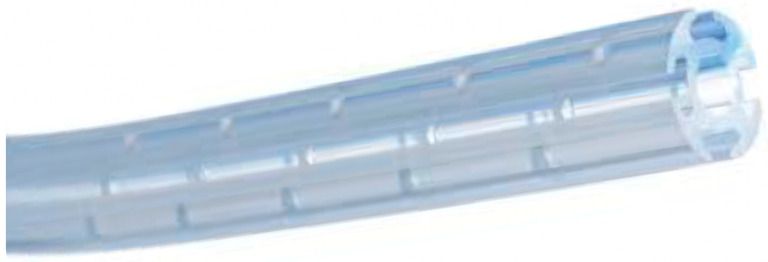
A smart coaxial drain structure with an internal lumen, four external fluted channels and distal bores.

The second solution was the adoption of a smart coaxial drain (Redax^®^, Modena, Italy), a novel type of drain with coaxial structure (Figure 2), to improve the drainage of air and fluid along the entire length of the tube, independently from its position and shape inside the thorax [16]. Indeed, the internal lumen of the tube with distal bores allows for effective air evacuation, while the four external fluted channels enable fluid drainage by capillarity (Figure 2).

However, the efficacy of both methods in uniportal VATS has not been proven yet and, most importantly, has never been compared with the insertion of the tube in a lower, more gravitationally influenced space, such as the VII/VIII one.

Therefore, the aim of this study is to evaluate whether the efficacy and safety of the chest tube are related to the level of its insertion through the intercostal space (IV/V as in uniportal VATS or VII/VIII as in biportal VATS) or to the specific type of drain employed (standard or smart coaxial drain).

## 2. Materials and Methods

### 2.1. Ethical Statement

The study is based on a prospective, multicenter, non-randomized trial approved by our Ethics Committee (Università Cattolica del Sacro Cuore, Rome) in March 2019 (Protocol 2900/19 ID:2388; ClinaclTrials.gov: NCT06036667) and, therefore, performed in accordance with the ethical standards of the Declaration of Helsinki and its later amendments. All patients signed informed consent for their participation in the study and for the anonymous treatment of their clinical data.

The Strengthening the Reporting of Observational Studies (STROBE) checklist was followed for reporting data and results in this study.

The centers involved were 3 European high-volume thoracic units with long experience in uniportal VATS (2 centers) and biportal VATS (1 center) surgeries.

The inclusion criteria were rigorously implemented in order to minimize any possible bias: only adult patients (age >18 years) who provided informed consent and who underwent upper pulmonary lobectomies (left or right) in uniportal or biportal VATS with lymphadenectomy.

The exclusion criteria were as follows: any type of associated lung or pleural resection; middle or lower lobectomies; previous thoracic surgery or radiotherapy; severe lung emphysema; severe adhesions discovered during surgery; patients discharged with a chest tube connected to a Heimlich valve (due to a persistently high amount of effusion or air leakage (>8 days)). All types of lobectomy except the upper one were excluded in order to reduce biases related to different potential residual empty spaces after surgery, which are known to be higher after upper lobectomies [12,13].

The results from patients enrolled from May 2019 to July 2023 were presented in this study.

### 2.2. Uniportal and Biportal VATS

All surgeries were performed under general anesthesia and single-lung ventilation, with patients in the lateral position.

In the uniportal VATS approach, the 4 cm incision was performed at the IV/V intercostal space along the middle axillary line [7,8,9]. The chest tube was inserted through the same incision as in Figure 1.

The type of chest tube used (28 Fr standard or smart coaxial) after the uniportal VATS upper lobectomies was determined by the models of tube available at the center during patients’ enrollment.

In biportal VATS, a utility incision measuring 4 cm was performed at the IV-V intercostal space along the middle axillary line. Additionally, a second 1 cm incision was made at the VII/VIII intercostal space (middle axillary line) for the camera port. At the end of surgery, the 28 Fr smart coaxial drain was introduced through this last incision, ensuring that its tip was positioned in the apex of the chest cavity.

Hilar and mediastinal lymphadenectomies were performed in all cases by utilizing energy devices (Maryland LigaSure^TM,^ Medtronic, Minneapolis, MN, USA) in order to reduce lymphatic or hematic leakage. The use of hemostatic agents and glues was at the discretion of the surgeon.

Therefore, according to the type of approach performed (level of intercostal space for chest drain insertion) and model of drain used, the patients were prospectively enrolled in the following 3 groups:

- The “U-VATS smart” (study group): uniportal VATS upper lobectomies with a 28 Fr smart coaxial drain inserted through the incision space at the end of surgery [7,8,9];
- The “U-VATS standard” (control group 1): uniportal VATS upper lobectomies with a 28 Fr standard drain inserted in an S-shaped manner through the incision space at the end of surgery;
- The “Bi-VATS smart” (control group 2): biportal VATS upper lobectomies with a 28 Fr smart coaxial drain inserted through the VII-VIII intercostal space at the end of surgery.

The study group U-VATS smart was compared independently with the two control groups, namely U-VATS standard and Bi-VATS smart, to evaluate any potential differences in primary and secondary outcomes.

The main clinical, surgical and radiological variables prospectively recorded per patient in the groups were as follows: sex; age; smoking habits; body mass index (BMI); chronic obstructive pulmonary disease (COPD); any cardiovascular disease; American Society of Anesthesiologists (ASA) score; tumor dimension; side of surgery; fissure-less lobectomy; number of lymph nodes retrieved; lung functionality (Pa0_2_, FEV1%, FVC%, DLCO); daily fluid output; residual effusion and pneumothorax (PNX) at last chest X-ray before chest tube removal; localization (anterior vs. posterior) and extension (measured in intercostal spaces on posterior arches); necessity of a second chest tube; mild subcutaneous emphysema; air leakage > 5 days; necessity for suction (−20 cm H_2_O); drainage blocked by clots at removal time; chest tube length; hospitalization (number of days from surgery to discharge); 30-day mortality; 90-day readmission for pleural effusion or PNX to be drained; post-operative pain (measured by visual analogue scale (VAS) score) during the first 2 post-operative days and at chest tube removal; and the onset of moderate/severe neuralgia 12 weeks after surgery.

### 2.3. Post-Operative Management

At the end of surgery, each patient underwent multilevel intercostal nerve block using 0.5% ropivacaine (4 cc in the incision space as well as in the spaces above and below it) and had a post-operative pain management regimen, which involved administration of paracetamol (1 g × 3/day) and tramadol (50 mg max 3/day) as “rescue” therapy.

In the post-operative period, air leakage was measured through a conventional water seal system (note that the precise quantification of air leakage was not the primary aim of this study). The application of suction (−20 cm H_2_O) to the chest tube was not a standard practice after surgery. Indeed, no suction was used unless it was deemed clinically necessary by thoracic surgeons (criteria for the use of suction: air leakage associated with moderate–severe subcutaneous emphysema or inadequate lung expansion with PNX > 4 intercostal spaces measured on the posterior arches of the ribs on chest X-rays). All patients underwent chest X-rays immediately after surgery and the day before drainage removal. If lung expansion was adequate, the chest tube was removed, provided there were no signs of air leakage for at least 24 h and fluid output was below 250 cc per day.

### 2.4. Primary and Secondary Outcomes

The primary outcomes were the amount of fluid output in each group during the first 3 post-operative days and overall.

The secondary outcomes were as follows: radiological findings (residual pleural effusion or PNX at chest X-ray); necessity of a second chest tube insertion in the post-operative period for clinically significant residual effusion (symptomatic or with a level over the posterior arch of the VI rib on a chest X-ray) or persistent PNX (more than 4 intercostal spaces measured on the posterior arches of the ribs on an X-ray after at least 24 h of suction); air leaks > 5 days; subcutaneous emphysema; post-operative complications; pain during the first 2 post-operative days (at rest and during coughing) and at chest tube removal; hospitalization and 90-day re-admission for pleural effusion or PNX to be drained (according to the same criteria described above for the necessity of a second chest tube during the post-operative period); the onset of moderate/severe neuralgia 12 weeks after surgery.

### 2.5. Sample Size

The trial was designed to test the hypothesis that the amount of pleural fluid after uniportal VATS upper lobectomy with a smart coaxial drain is superior (based on a previous study in triportal VATS and open surgery [17,18]) to uniportal VATS with a standard drain.

An “A priori” analysis was performed given an effect size of 0.4, a power of 90% and a type I error of 5% (α). A 10% dispersion of patients was also considered. Therefore, 59 patients per group were estimated. G*Power was used for sample size calculation [19].

### 2.6. Statistical Analysis

The categorical variables were expressed as numbers (%), and the continuous variables were expressed as mean ± standard deviation. The categorical variables were compared using a chi-squared test or Fischer’s exact test; the continuous variables were compared using the independent sample Student’s *t*-test or the Mann–Whitney U-test, if normally or non-normally distributed.

A *p* < 0.05 was considered statistically significant.

A statistical analysis was performed using IBM SPSS Statistics for Macintosh, version 25.00 (Armonk, NY, USA).

## 3. Results

**Figure 3:**
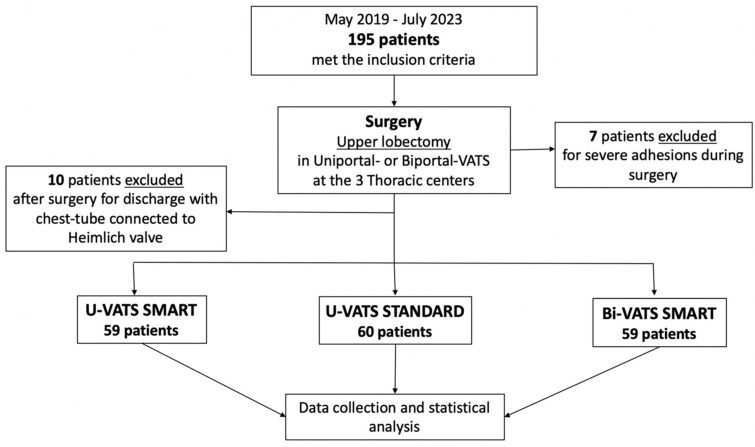
Flow diagram of the study.

From May 2019 to July 2023, 195 patients met the inclusion and exclusion criteria in the three thoracic centers and were prospectively enrolled in the trial. Seven patients were excluded at the time of surgery due to severe pleural adhesions (strong adhesions involving the whole lung surface, including the residual lobes, and causing disruption of the visceral and/or parietal pleural during the dissection attempt). Ten patients were excluded after surgery because they were discharged with a chest tube in place connected to a Heimlich valve, which was removed after a median of 12 days after surgery (Figure 3).

Only 178 patients completed the trial: 59 in the U-VATS smart group, 60 in the U-VATS standard and 59 in the Bi-VATS smart group.

Comparing the study group U-VATS smart with one of the control groups, U-VATS standard, both groups presented comparable baseline clinical and surgical characteristics (Table 1).

**Figure 4:**
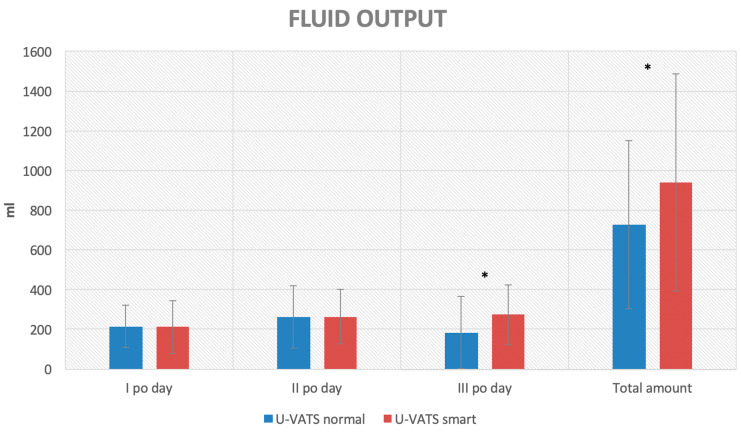
Column chart of the primary outcome for the U-VATS smart vs. U-VATS standard group comparison (* = *p* < 0.05).

Based on the primary outcomes, a higher fluid output was recorded in the U-VATS smart group (274 ± 150 vs. 183 ± 182 mL, *p*: 0.004) in the III p.o. and overall (941 ± 547 vs. 725 ± 423 mL, *p*: 0.027) compared to the U-VATS standard one (Figure 4).

Concerning secondary outcomes, there was no difference between the two groups in terms of all parameters measured, radiological findings, hospitalization, level of post-operative pain, incidence of chronic moderate/severe neuralgia, etc. (Table 2).

Only a higher 90-day readmission due to residual pleural effusion to be drained (effusion level over the posterior arch of the VI rib on chest X-ray) in U-VATS standard (four (6.6%) vs. zero, *p*: 0.04) was recorded.

In particular, it should be noted that all other cases of residual effusions in both groups (Table 2) were mainly anterior and clinically not significant (under the criteria established above for a second chest tube insertion), such as PNX (33.3% in U-VATS standard and 30.5% in U-VATS smart, *p*: 0.959) that had always a mean extension of 1.22 ± 1.27 intercostal spaces in U-VATS standard vs. 1.00 ± 1.15 in U-VATS smart (*p*: 0.405).

In terms of baseline characteristics (Table 3), the study group U-VATS smart was also comparable to the other control group, Bi-VATS smart, with the exception of a higher number of smokers (*p*: 0.03).

**Figure 5:**
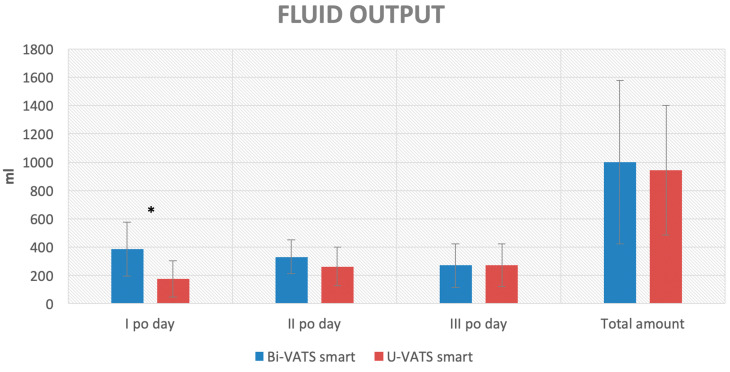
Column chart of the primary outcome for the U-VATS smart vs. Bi-VATS smart group comparison (* = *p* < 0.05).

Comparing U-VATS smart with Bi-VATS smart, a higher fluid output was recorded in the I post-operative day in Bi-VATS smart (385 ± 189 vs. 178 ± 128 mL, *p* < 0.001), with no difference in the other post-operative days or in the total amount of fluid (Figure 5).

Regarding secondary outcomes, a higher incidence of residual effusion on the final chest X-ray was observed in U-VATS smart (eight patients (13.5%) vs. zero, *p*: 0.004), and it was localized anteriorly in all cases. An increased prevalence of residual PNX was also recorded in U-VATS smart compared to Bi-VATS smart (18 patients (30.5%) vs. 2 (3.4%), *p*: <0.001), and it was extended in mean for 1.00 ± 1.15 intercostal spaces vs. 0.15 ± 0.50 intercostal spaces (*p*: <0.001), Table 4. None of the cases in any group required an additional chest tube during the post-operative period. A higher number of mild subcutaneous emphysemas was observed in U-VATS smart (15 cases (25.4%) vs. 6 (10.2%), *p*: 0.002), with no difference in the use of suction in both groups (*p*: 0.765). No patient was readmitted for residual PNX or effusion to be drained 90 days after surgery, regardless of the group.

There was no difference in terms of pain on the I and II post-operative days, with the exception of a higher VAS score during coughing in Bi-VATS smart on the II post-operative day (0.03). A higher incidence of moderate/severe neuralgia 12 weeks after surgery was also documented in Bi-VATS in comparison with U-VATS smart (six (10.2%) vs. one (1.7%), *p*: 0.03), Table 4.

## 4. Discussion

In the era of less invasiveness and enhanced recovery after surgery (ERAS), the traditional double drainage system following pulmonary lobectomy has been progressively substituted by a single chest tube inserted in the lower part of the chest cavity. In particular, in VATS lobectomy, the use of a single chest tube has become the standard in the last few years [2,3,4] in order to maximize the benefits of minimally invasive surgery and reduce post-operative pain, improving patients’ recovery. The safety and effectiveness of a single chest tube in triportal and biportal VATS lobectomies are already proven [3,4]; however, the question is still open and debated in uniportal VATS. The main concern is that, due to the higher location of the single incision in uniportal VATS (IV-V intercostal space), the chest tube inserted through this space may not provide adequate fluid, air drainage and lung expansion, particularly after upper lobectomies [13]. Several authors provided possible solutions, evaluating the efficacy of the use of a modified T-tube [15] or an additional pleural catheter compared to a standard chest tube after uniportal VATS lobectomy [12,13].

To the best of our knowledge, this is the first prospective multicenter trial that aims to answer the question of whether the efficacy of a chest tube in uniportal VATS lobectomy is related to the drain type (standard vs. coaxial smart) or to the level of insertion (IV/V space vs. VII/VIII as in biportal VATS) without using additional drainage, a pleural catheter [12,13] or a pigtail [20].

As stated before, it is fundamental to mention that the suggested position of the standard chest tube in uniportal VATS, based on our experience (started in 2016), is as shown in Figure 1 in order to maximize fluid and air output.

The proposed use of the other innovative type of drain, the coaxial smart drain, has never been tested before in uniportal VATS. Its efficacy was already demonstrated following lobectomy in thoracotomy [16], where the coaxial drain appeared to be not inferior to the standard double chest tube drainage in effectiveness while also resulting in shorter hospital stays and less pain.

According to our results, the smart coaxial drain compared to the standard drain in uniportal VATS appeared to enhance the total fluid volume drainage, with no significant differences in radiological findings (residual PNX or effusion), subcutaneous emphysema, hospitalization or acute and chronic pain. Only a significantly higher readmission was recorded in the group of patients who underwent uniportal VATS upper lobectomy using a standard 28 Fr tube (four (6.6%) vs. zero, *p*: 0.04) for a residual pleural effusion that required drainage.

The same smart coaxial drain, if inserted through the VII/VIII intercostal space, as in biportal VATS, rather than the IV/V space (uniportal VATS), did not increase the total amount of fluid drained (Figure 5) but recorded a higher incidence of acute pain during coughing in the II p.o. (*p*: 0.03) and chronic moderate/severe neuralgia (*p*: 0.03, Table 4). Although the smart coaxial drain is considered to be softer than standard tubes, a higher level of acute and chronic pain was recorded in the biportal VATS group that could be related to the position of the insertion of the tube itself. Indeed, the VII intercostal space is used during surgery in biportal VATS for introducing the camera through a thoracoscopic rigid port. The movements of the camera in its port during surgery may increase the risk of intercostal nerve injury and consequent neuralgia in the post-operative period [21]. Furthermore, the lowest intercostal spaces (like the VII/VIII spaces used in Bi-VATS) are often narrower than the IV or V intercostal spaces, above all in women, and this fact may increase the risk of nerve compression by the camera port during surgery or by the chest tube after surgery. Also, in our opinion, the most inferior location of the chest tube in Bi-VATS may cause some diaphragmatic irritation in the patient during deep breathing and coughing.

It is worth noting that, in order to reduce biases in the evaluation of chronic pain onset as well as the long duration of drainage, patients discharged with a chest tube connected to a Heimlich valve were excluded, and no difference in chest tube length was registered among the two groups (*p*: 0.222, Table 4).

Patients who underwent uniportal VATS upper lobectomies with the smart coaxial drain had a higher incidence of residual PNX at the final chest X-ray (*p*: <0.001), with a mean extension of 1.00 ± 1.15 intercostal spaces, and a higher incidence of residual effusion (*p*: 0.004) localized anteriorly compared to patients who underwent biportal VATS with the smart coaxial drain. In no case were these radiological findings clinically significant enough to require the insertion of an additional chest tube in the post-operative period.

Therefore, the main advantage related to the use of a smart coaxial drain, confirmed in this study, is that it can drain air and fluid along its entire length even when inserted through the IV-V intercostal space (as in uniportal VATS) and independently from its position and shape inside the thorax. This makes the smart coaxial drain a good solution for inexperienced uniportal VATS surgeons or in all those cases where it may be difficult to insert the chest tube through a uniportal VATS incision with the suggested “S-shape” due to the diameter of the standard tube itself, the clinical characteristics of the patients (obese patients, small or huge thoraxes) or the type of lung resection (wide lung resections with huge residual empty spaces, etc.). This means that some surgeons may prefer to place the drainage through an additional incision in the VII/VIII space or by using an additional catheter, as suggested in other studies [12,13,20], to reduce potential risks or residual effusion. In all of these situations, the adoption of a smart coaxial drain in uniportal VATS could improve the drainage of pleural space, solving the problem of clinically significant residual pleural effusion or PNX, while preserving the minimally invasiveness of the single access in terms of less post-operative and chronic pain, as well as yielding better cosmetic results. Indeed, the smart drain is softer and more convenient to adjust in the desired shape inside the chest cavity, and, even in the case of malposition of the smart tube itself, it can still ensure drainage along its whole surface by capillarity. It should also be noted that the enrollment of only upper lobectomies (where the potential risk of an empty pleural cavity in the apex is known to be greater) maximizes the results of this study while reducing potential confounders.

The potential drawback associated with the use of a smart coaxial drain could be the cost, which is about three times that of a standard chest tube (about 65 euros vs. 22 euros, [16]); however, this can be well balanced out by the non-necessity for a second chest tube/pleural catheter, less chronic neuralgia (with less chronic consumption of painkillers), faster patient recovery and a lower risk of potential readmission for residual pleural effusion to be re-drained.

### Limitations and Points of Strength

This study has some limitations and points of strength. First of all, it was a non-randomized trial.

However, a notable aspect of strength was the involvement of three European centers with extensive experience in uniportal and biportal VATS, and strict criteria were respected for the enrollment of patients (only upper lobectomies without any other associated lung or pleural resection, etc.) in order to reduce any potential bias.

The enrollment was prospective, and the estimated sample size was reached in all groups.

Although it was not possible to completely standardize some clinical practices across the centers, some rules were respected for the use of suction in the post-operative period or the evaluation of radiological findings on chest X-rays. Indeed, the decision regarding the use/timing of suction prescriptions was at the surgeon’s discretion but in accordance with some clinical parameters described above. Then, the use of chest X-rays was not completely standardized across all the centers; thus, only the findings on the final X-ray, before chest tube removal, were evaluated in order to have the same radiological criteria of evaluation in all centers based on a two-projection radiograph and not in the supine position (as it might have been in the case of the first post-operative X-ray). Lastly, this is the first prospective multicenter trial that aims to evaluate the safety and efficacy of chest tubes in uniportal VATS lobectomies.

## 5. Conclusions

Standard chest tube insertion through the same incision space in uniportal VATS appears to be safe and effective, provided that specific precautions and suggestions are duly respected.

The use of a smart coaxial drain has the potential to enhance fluid output in uniportal VATS, mimicking the insertion of drainage in a lower space (i.e., biportal VATS), thereby reducing the potential risk of 90-day readmission due to residual pleural effusion to be drained, but with less pain and incidences of chronic neuralgia.

## Figures and Tables

**Table 1:** Comparison of the main clinical and surgical characteristics of patients in the U-VATS smart group and the U-VATS standard group.

	U-VATS Smart (n° 59)	U-VATS Standard (n° 60)	*p*
Sex (male)	28 (47.5%)	28 (46.7%)	0.939
Age (years)	67.65 ± 9.19	69.62 ± 6.68	0.140
Smoker	15 (25.4%)	19 (31.6%)	0.872
BMI	25.90 ± 3.42	26.16 ± 5.29	0.795
COPD	18 (30.5%)	23 (38.3%)	0.450
Cardiovascular diseases	37 (62.7%)	43 (71.7%)	0.399
Side (Right)	35 (59.3%)	41 (68.9%)	0.392
Tumor dimension (cm)	2.35 ± 0.98	2.71 ± 1.4	0.103
Fissureless lobectomy (stapler)	50 (85.0%)	53 (88.3%)	0.404
Lymph nodes retrieved	12.32 ± 7.45	14.56 ± 8.75	0.658
PaO_2_	81.04 ± 15.48	76.54 ± 15.41	0.446
FEV1%	98.50 ± 19.13	90.50 ± 27.61	0.398
FVC%	104.67 ± 6.97	111.36 ± 22.73	0.407
DLCO	45.11 ± 42.64	36.28 ± 39.12	0.716

**Table 2:** Results of group U-VATS smart compared with U-VATS standard.

	U-VATS Smart (n° 59)	U-VATS Standard (n° 60)	*p*
Residual effusion at last chest X-ray	9 (15.3%)	12 (20.3%)	0.289
Anterior residual effusion	8 (13.5%)	9 (15.0%)	0.594
Residual PNX at last chest X-ray	18 (30.5%)	20 (33.3%)	0.959
Residual PNX (number of intercostal spaces)	1.00 ± 1.15	1.22 ± 1.27	0.405
Necessity for second drainage insertion	0	0	1.00
Air leakage > 5 days	4 (6.8%)	4 (6.7%)	0.744
Mild subcutaneous emphysema	15 (25.4%)	12 (20.0%)	0.581
Necessity for suction (−20 cmH_2_O)	13 (22.0%)	9 (15.0%)	0.414
Drainage blocked by clots at removal	0	1 (1.6%)	0.343
Chest tube length	4.20 ± 1.34	4.05 ± 1.16	0.573
In-hospital stay	4.20 ± 1.04	4.29 ± 1.36	0.738
30-day mortality	0	0	1.00
90-day readmission for effusion to be drained	0	4 (6.6%)	0.04 *
90-day readmission for PNX to be drained	0	1 (1.7%)	0.310
VAS scale I p.o. day at rest	3.50 ± 1.93	3.16 ± 1.35	0.351
VAS scale I p.o. day during coughing	3.50 ± 1.93	4.27 ± 1.50	0.351
VAS scale II p.o. day at rest	4.71 ± 1.97	2.75 ± 1.43	0.258
VAS scale II p.o. day during coughing	3.13 ± 1.77	3.36 ± 1.54	0.285
VAS scale at chest tube removal	3.97 ± 1.83	2.82 ± 1.50	0.123
Chronic moderate/severe neuralgia	3.05 ± 1.08	2 (4.5%)	0.470

* *p* < 0.05.

**Table 3:** Comparison of the main clinical and surgical characteristics of patients in the U-VATS smart group and the Bi-VATS smart group.

	U-VATS Smart (°59)	Bi-VATS Smart (n° 59)	*p*
Sex (male)	28 (47.5%)	29 (49.2%)	0.833
Age (years)	67.65 ± 9.19	68.79 ± 8.34	0.09
Smoker	15 (25.4%)	27 (45.8%)	0.03 *
BMI	25.90 ± 3.42	25.24 ± 5.40	0.533
COPD	18 (30.5%)	27 (45.8%)	0.173
Cardiovascular diseases	37 (62.7%)	34 (57.6%)	0.193
Side (Right)	35 (59.3%)	41 (69.5%)	0.389
Tumor dimension (cm)	2.35 ± 0.98	2.14 ± 1.48	0.312
Fissureless lobectomy (stapler)	50 (85.0%)	38 (64.4%)	0.08
Lymph nodes retrieved	12.32 ± 7.45	14.00 ± 10.65	0.09
PaO_2_	81.04 ± 15.48	79.83 ± 18.21	0.274
FEV1%	98.50 ± 19.13	91.60 ± 19.84	0.132
FVC%	104.67 ± 6.97	96.20 ± 12.63	0.105
DLCO	45.11 ± 42.64	48.45 ± 30.78	0.129

* *p* < 0.05.

**Table 4:** Results of group U-VATS smart compared with Bi-VATS smart.

	U-VATS Smart (n° 59)	Bi-VATS Smart (n° 59)	*p*
Residual effusion at last chest X-ray	9 (15.3%)	0	0.004 *
Anterior residual effusion	8 (13.5%)	0	0.05 *
Residual PNX at last chest X-ray	18 (30.5%)	2 (3.4%)	<0.001 *
Residual PNX (number of intercostal spaces)	1.00 ± 1.15	0.15 ± 0.50	<0.001 *
Necessity for second drainage insertion	0	0	1.00
Air leakage > 5 days	4 (6.8%)	3 (5.1%)	0.891
Mild subcutaneous emphysema	15 (25.4%)	6 (10.2%)	0.02 *
Necessity for suction (−20 cmH_2_O)	13 (22.0%)	12 (20.3%)	0.765
Drainage blocked by clots at removal	0	0	1.00
Chest tube length	4.20 ± 1.34	3.76 ± 1.90	0.222
In-hospital stay	4.20 ± 1.04	4.94 ± 2.05	0.141
30-day mortality	0	0	1.00
90-day readmission for effusion to be drained	0	0	1.00
90-day readmission for PNX to be drained	0	0	1.00
VAS scale I p.o. day at rest	3.50 ± 1.93	3.47 ± 2.53	0.816
VAS scale I p.o. day during coughing	4.71 ± 1.97	5.06 ± 2.05	0.477
VAS scale II p.o. day at rest	3.13 ± 1.77	3.29 ± 1.90	0.547
VAS scale II p.o. day during coughing	3.97 ± 1.83	5.29 ± 2.17	0.03 *
VAS scale at chest tube removal	3.05 ± 1.08	3.53 ± 2.07	0.527
Chronic moderate/severe neuralgia	1 (1.7%)	6 (10.2%)	0.03 *

* *p* < 0.05.

## Data Availability

The data presented in this study are available on request from the corresponding author.
